# Proficiency Barrier in Track and Field: Adaptation and Generalization Processes

**DOI:** 10.3390/s24031000

**Published:** 2024-02-04

**Authors:** M. Teresa S. Ribeiro, Filipe Conceição, Matheus M. Pacheco

**Affiliations:** 1 Research Center in Sport Sciences, Health and Human Development (CIDESD), Physical Education and Sport Sciences Department, University of Maia, 4475-690 Maia, Portugal; teresaribeiro@umaia.pt; 2 Center for Investigation, Formation, Innovation and Intervention in Sports, Faculty of Sport, University of Porto, 4200-450 Porto, Portugal; filipe@fade.up.pt; 3 GEDEM, Department of Physical Education, Federal University of Rondônia, Porto Velho 78900-000, Brazil

**Keywords:** transfer of performance, motor development, stages, skill acquisition

## Abstract

The literature on motor development and training assumes a hierarchy for learning skills—learning the “fundamentals”—that has yet to be empirically demonstrated. The present study addressed this issue by verifying (1) whether this strong hierarchy (i.e., the proficiency barrier) holds between three fundamental skills and three sport skills and (2) considering different transfer processes (generalization/adaptation) that would occur as a result of the existence of this strong hierarchy. Twenty-seven children/adolescents participated in performing the countermovement jump, standing long jump, leap, high jump, long jump, and hurdle transposition. We identified the proficiency barrier in two pairs of tasks (between the countermovement jump and high jump and between the standing long jump and long jump). Nonetheless, the transfer processes were not related to the proficiency barrier. We conclude that the proposed learning hierarchy holds for some tasks. The underlying reason for this is still unknown.

## 1. Introduction

In the motor development and training literature (e.g., [1,2,3,4]), there is a long-standing assumption that maintains a high priority for learning the “fundamentals”. In general terms, this is the assumption that simpler or basic skills must be practiced and learned before one can further specialize in or learn more complex skills. Examples of this assumption are found in descriptive models and guidelines in sports/physical education (see, for instance, [5]).

These fundamentals are usually related to two different ideas, depending on the area. In motor development, a strong case is made for the “fundamental movement patterns” [6]. The fundamental movement patterns are broadly defined [7] as those performed in “common” motor activities without imposed specific performance goals. Examples of these common activities are throwing, kicking, and running. In these descriptive models, it is implied that one would be unable to learn sport-specific movement patterns (e.g., dart throwing, specific kicking patterns in soccer, and 100 m sprint running) if these fundamental skills are not being performed skillfully [1,8,9].

In training, the expected relationship between fundamentals and performance is more subtle. Usually, the claim is in terms of a given physical ability (e.g., “reactive force”, “explosive strength”, and “agility”) that must be developed so one can demonstrate good performances of sport-specific movement patterns (see [3]). Note, nonetheless, that the discussed physical ability is usually considered (and measured) within the context of a given “basic” skill (e.g., explosive strength can be measured by the countermovement jump). In some cases, the basic skill is used for practice, as it would also be the best way to develop such a physical ability.

In both cases, there is an implied hierarchy of the learning contents for a learner to perform well in sport skills. Vern Seefeldt [1,8] promoted a strong case for this hierarchy: he stated that those with low proficiency (a large difference from a (“gold”/optimum) standard movement pattern) in the fundamental movement skills would have great difficulty in learning more complex skills in motor development. He termed this considerable difficulty the “proficiency barrier”. It should be emphasized that this hypothesis is a direct implication from many of the stage models of motor development [1,2,5,10] that encompass a hierarchy of learning.

In recent years, a debate has started concerning the ontological status of these fundamentals. Newell [11], for example, questioned whether the so-called fundamental movement skills meet the necessary conditions to be called “fundamental” to start with. Others have tried to provide new terminology (calling them foundational movement skills [12]), calling into question their centrality in developmental theories [13] or defending their centrality in motor development [6]. 

Notably, one still needs to demonstrate empirical evidence on the proposed hierarchy involving these skills (or the assumed hierarchy). Only recently did three studies directly support the proficiency barrier hypothesis [9,14,15]. These studies considered whether superior results in two fundamental movement patterns’ assessments (e.g., running and bouncing a ball) were necessary for either demonstrating or learning a more complex (sport-specific) movement pattern that is supposed to be a combination of two simpler ones (e.g., dribbling). Nonetheless, it is not true that the hierarchy holds for all situations. The aforementioned supportive results contrast with other studies that failed to show such a dependency [16,17]. In the contrasting studies, either one could learn a sport-specific skill without showing good results in the fundamental skills [17] or the relationship among skills existed but showed no signs of a given proficiency barrier [16].

There are many potential reasons why the proficient barrier does not hold for all cases. The main one, we argue, is that one can expect more than a single relationship between skills: generalization and adaptation. Both can be said to be types of transfer (i.e., the effect that practice in task A has on performance of task B [18]). Generalization is simply the application of something learned in a specific context to others. This is usually what is supposedly studied in motor learning experiments when researchers implement experimental designs with transfer tests of 10 to 20 trials after practice (also, see the rationale behind some theories [19,20]). Adaptation, on the other hand, is a process of change that occurs *based* on what was learned in the original practice. This is what is usually implied in motor development—what can be learned is dependent on the previous experiences/tendencies of the individual (see, for instance, [21,22]). Few studies have used such terminology, but they demonstrated this process (see [23,24] for an approach based on such a process). Importantly, studies have only considered one or the other in their designs and, for this reason, the principles that differentiate when one or the other is observed are unexplored.

Our main purpose is to address a long-standing assumption on the necessity of learning fundamental movement patterns for learning sport-specific skills and the processes that would be based on such a necessity. Specifically, we investigate whether the appearance of a proficiency barrier is a matter of the type of transfer that occurred. Our hypothesis is that the proficiency barrier occurs when adaptation is required. This would be the case when what is learned in the fundamental skill is not what is performed for the sport skill but a necessary condition for the sport skill to be learned. Only after learning the required (i.e., fundamental) skill “components” can the more complex skill be learned, i.e., a nonlinear relationship (such as in [14,15]). On the other hand, if one can generalize learning from the fundamental skill to the sport skill, then the better one is at the fundamental skill, the better one is at the sport skill, i.e., a linear relation (such as in [16]).

To reach our goal, the present study investigates how three “fundamental” skills relate to three sport-specific skills in track and field. We selected the countermovement jump, the horizontal jump, and the obstacle jump as fundamental movement skills and the high jump, long jump, and hurdles as sport-specific skills. To investigate the relationships among the skills, we (1) assessed the potential existence of a proficiency barrier and (2) verified whether the proficiency barrier relates to generalization/adaptation processes. Different from previous studies on the topic [9,14,15,16] that implemented checklist-based assessments, we analyzed the hypothesis through motion tracking technology to identify movement pattern characteristics that differentiate individuals without an a priori assumption of them.

## 2. Methods

### 2.1. Sample

Twenty-seven young athletes (15 girls) participated in this study. Table 1 shows the sample’s characteristics. The participants were, at that time, affiliated with a track and field outreach program of the Faculty of Sport. Participation in this study was voluntary. Legal guardians read and signed an informed consent form. The Ethics Committee of the Faculty of Sport, University of Porto, approved all procedures.

### 2.2. Task and Materials

To characterize participants’ anthropometry, we used a stadiometer (Seca 213 stadiometer, Hamburg, Germany) with a precision of 0.1 cm and a portable bioimpedance scale (Tanita BC-730, Tokyo, Japan). All measurements adhered to the protocols established by the International Working Group on Kineanthropometry standards [25].

**Figure 1:**
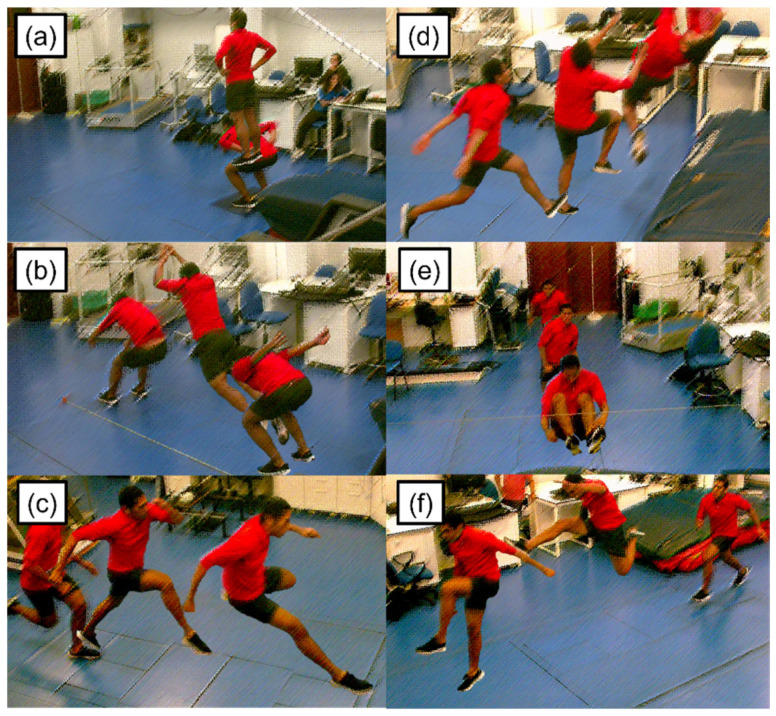
Exemplary participant performing the (**a**) countermovement jump; (**b**) standing long jump; (**c**) leap; (**d**) high jump; (**e**) long jump; and (**f**) hurdle transposition.

Participants performed six tasks. Figure 1 shows the countermovement jump, standing long jump, leap, high jump, long jump, and hurdle transposition. The order of data collection was always the same for all participants: countermovement jump, standing long jump, leap, long jump, high jump, and hurdle transposition. We considered the first three tasks as “fundamental” in the sense that they are performed as general activities (not necessarily in the context of sports). The last three tasks are specific events in track and field (representing, thus, the sport-specific assessment). Eight motion capture cameras recorded all tasks at 100 Hz (Miqus Video, Qualisys AB, Gothenburg, Sweden). For all tasks, participants performed ten repetitions with a 30 s rest interval. 

For the countermovement jump, the athlete starts in a standing position with feet parallel and shoulder-width distance. After the experimenter commands “go”, the athlete was instructed to jump as high as possible after a fast flexion of the hips and knees. 

In the standing long jump, the athlete begins in a standing position, with feet parallel and behind the starting line. The experimenter instructed the athlete that, upon the “go” signal, he/she should jump as far as possible forward. The jumps were measured with a measuring tape placed parallel to the jumping direction.

For the leap, we implemented an adapted version of the leap used in the Test of Gross Movements Development (TGMD-2 [26]). The experimenter instructed the athlete to run toward a “water puddle” and leap over it. Also, the athlete was supposed to continue to run after landing.

For the high jump test, the experimenter instructed participants to run and jump with their preferred foot, aiming to jump as high as possible (over an “imaginary” bar) and then land with their back on the mattress. If the preferred foot was not known, a few familiarization trials were permitted.

In the long jump test, participants were instructed to run and jump with their preferred foot, aiming to jump as far as possible and then land in a seated position. The landing was performed on a suitable mattress for this purpose.

For the hurdle transposition, the experimenter instructed athletes to run and jump over an imaginary hurdle (clear the hurdle) placed in the middle of the running path. After clearing the hurdle, the athletes were instructed to continue running.

### 2.3. Procedures

Each evaluation session was carried out with a single participant and in the same place (University of Porto Biomechanics Laboratory). Before each session, the space was calibrated while participants performed a warm-up (slow run and mobility exercises). Then, anthropometric measures were taken, and the participant received instructions on each motor task to be performed. For all tasks, the experimenters provided no demonstration to the participant beyond the general instructions described in the previous session. 

### 2.4. Data Analysis

After data collection, we used Theia3D (v2023.1.0.3161, Theia Markerless, Inc., Kingston, ON, Canada) to extract the joint motion of each participant. Theia3D offers a markerless motion capture solution that relies on synchronized video data to generate precise and dependable 3D pose estimations of human subjects visible in the footage. The system employs advanced deep learning algorithms (deep convolutional neural networks) trained to recognize humans and accurately predict the 2D positions of over 100 landmarks on the human body for each frame in every camera’s video. By applying a subject-specific inverse kinematic model scaled to the predicted landmarks, the 3D pose of the human is reconstructed and continuously tracked throughout their movements. This data-driven approach ensures a robust solution that is applicable across various environments and movements, enabling the efficient collection of high-quality 3D motion capture data. Theia3D has shown higher reliability than marker-based methods for lower limb kinematics (less than 3.5° of variability) [27] and, given the complexity of the upper limb kinematics, an error in the range of 8.1 to 23° for the upper limb joints (root mean squared error) [28].

The data were processed considering arms and feet as 6-degrees of freedom segments and with a low-pass filter at 20 Hz. The data were further processed using a designed Matlab script (all codes can be assessed in https://osf.io/bgxa8/, accessed on 3 February 2024) (Matlab R2023b Update 4 [23.2.0.2428915]). 

For all joint motion analyses, 20 dimensions were considered: ankle flexion, knee flexion, hip flexion and abduction, thorax flexion, abduction and rotation, shoulder flexion and abduction, elbow flexion, and pelvis angle relative to the lab. We also considered some position measures of the center of mass, left foot center of gravity, and right foot center of gravity. 

For all joint motions, we shifted the angles that showed discontinuities around ±180°. Then, we filled the potential missing data within a trial with the *spline* function in Matlab and filtered the data with a 10 Hz fourth-order Butterworth low-pass filter. 

Provided some issues with near-static moments of the trial, the Theia3D software would create spurious 360° rotations around a given joint. The designed script would identify joint angles with rotations above 300° and identify the moment of rotation using the *findchangepts* function in Matlab (with a maximum of 2 changes: beginning and end of the spurious rotation). For trials in which this spurious rotation took less than 25% of the trial, the script cleared the frames in which the spurious rotation occurred, decreased the time series after the rotation by adding ±360° (depending on the direction of the spurious rotation), and filled the data with the *spline* function. Trials in which the spurious rotation took longer than 25% of the trial were not further considered in the analysis. This was not considered for pelvis rotations with reference to the laboratory (as this could occur in the high jump).

Considering potential issues in the Theia3D processing (such as not processing a given trial) or the aforementioned spurious rotations, we missed 48 trials (2.96% of the trials).

Before calculation of the performance measures or the movement patterns, we selected only the moment of interest in the whole recording. For the countermovement jump, we considered, as the beginning of the trial, the moment of the lower center of mass height before the center of the mass peak height and, as the end, the moment of the center of mass peak height. For the standing long jump, we considered, as the beginning of the trial, the moment of the maximum knee flexion before the peak velocity forward of the center of mass and, as the end of the trial, the first negative peak acceleration of the center of mass after the peak velocity forward. For the leap, long jump, and the hurdle transposition, as the beginning and end of the trial, respectively, we found the minima before and after the peak height of the center of mass. For the high jump, we considered, as the beginning of the trial, the second minimum before the center of the mass peak height and, as the end of the trial, the first minimum after the center of the mass peak height.

For all further analyses, the trials were time-normalized using the *spline* function in Matlab. Thus, all trials have 100 frames (1 to 100%).

#### 2.4.1. Performance

The performance of each task was derived according to the task’s demands. For the countermovement jump, we used the peak height of the center of mass as the performance. For the standing long jump, we used the landing distance of the individual. If the individual fell, we considered the local where the feet first touched the ground after the jump. For the leap, high jump, and hurdle transposition, we considered performance as the highest height the body achieved in the trial. For this, we considered the height of both the feet and center of mass over time and selected the lowest height “segment” over time. From this, we determined the maximum that the lowest height segment reached. For the long jump, we considered the distance reached derived from the velocity of the center of mass after the jump.

#### 2.4.2. Movement Patterns

To characterize the movement pattern, we performed a principal component analysis for each trial using all 20 joint motions. From the outcome, we considered the first 2 principal component coefficients (accounting for, on average, 89.78% of the variance of the data) and compared them among skills using the normalized dot product. We also calculated the cumulative sum of variance accounted for each principal component and noted how many principal components were necessary to explain at least 90% of the data.

#### 2.4.3. General Associations

As we have a large variety of ages and anthropometric characteristics, as well as six motor tasks being performed, we decided to characterize in general terms the associations among all variables before delving into our primary questions. For this reason, we first performed seven linear mixed effect models with performance outcomes for each condition as dependent variables and sex, age, fat percentage, and trials as independent variables. 

For the number of components, we used the average number per condition per participant and performed a Friedman’s ANOVA to understand whether these different tasks had a tendency toward qualitatively different movement patterns.

Additionally, as specific pairings were considered for the proficiency barrier and transfer processes assessments (see below), we determined the spearman’s *ρ* correlations among all six tasks, for the sake of completeness.

#### 2.4.4. Proficiency Barrier Assessment

The proficiency barrier is a phenomenon that is, primarily, tested longitudinally: if someone demonstrates low proficiency in a fundamental skill, the learning (a longitudinal process) of the sport skill becomes difficult, if not impossible. Pacheco et al. [15] pointed out that such a longitudinal process shows its signature in cross-sectional measurements and demonstrated it through a sigmoidal relationship between fundamental and sport skills. 

It is important to note, however, that the relationship is demonstrated among the skills’ movement patterns on a continuous scale. Despite the fact that movement patterns are categorical (i.e., one movement pattern is not intrinsically comparable to another by itself), studies got around this issue by employing criteria-based movement assessments. One can sum the achieved criteria for each skill and then see whether the relationship fits the expectation.

There are issues with these criteria-based assessments, nonetheless. First, this type of assessment implies that there is a given standard (i.e., a “champion model”) that the performer must follow. To our knowledge, this is hardly justifiable a priori: there is no reason to believe that individuals, with their own previous experiences and biomechanical individualities, converge to the same optimal technique (see [29,30]). Further, it implies a *unique* pathway between fundamental and sport skills learning: only if the athlete performs the fundamental movement pattern in the way that the assessment considers, then the athlete can learn the sport skill. It also assumes that the movement pattern is sufficient for measuring skill level. As numerous studies argue (see [31,32]), an athlete can reach a high level of performance through different movement patterns. The second issue is that, even if there was a single movement pattern standard for fundamental and sport skills, one would need to validate this type of assessment for all skills in order to understand the phenomenon at stake here. Considering the two issues together creates an insurmountable problem.

Thus, to infer a proficiency barrier between the fundamental tasks and the sport-specific ones, we performed a two-step procedure. First, we performed the same procedure as in Pacheco et al. [15]: we compared the linear and sigmoid functions fit between fundamental and sport-specific performances. The rationale behind the comparison is that, if there is a minimum value of a fundamental skill performance on which an individual must demonstrate to learn a sport skill, then the curve between the fundamental skill and sport skills performance would be nonlinear: two relationships (i.e., regimes) separated by a threshold value (i.e., the proficiency barrier). Pacheco tested two different possibilities (piecewise and sigmoid functions) and the sigmoid function demonstrated the best fit.

For this, we compared the resultant corrected Akaike Information Criterion (AICc, see [33]) between the linear function

S = α + β FMS(1)

and the sigmoidal function

S = α + (β − α)/(1 + exp(−δ*(FMS − γ))),(2)

where S is the sport-specific task and FMS is the fundamental movement skill; and α, β, δ, and γ are free parameters. The AICc penalizes the number of required parameters provided the explained variance of the fit—also being appropriate for small sample sizes. For interpretation, smaller AICc values refer to a better fit.

In Pacheco et al. [15], for the S and FMS in the above functions, they used a score of the summed criteria achieved by the performance of the movement pattern. Given the lack of such measures here, we fitted the function considering the performance average for each task.

The sigmoidal fitting was performed with the nonlinear least squares method using the *fit* function from Matlab R2023b. The free parameters α and β were constrained to have minima and maxima values of 10% (of the range) below and above, respectively, the S variable values considered in the pairing, and δ was constrained from 0 to infinity and γ from 10% below up to 10% above the FMS variable values. The starting points were considered the 25th percentile of the S variable values, the 75th percentile of the S variable values, 1, and the median of the FMS variable values for α, β, δ, and γ. The linear function was fitted using the same function with the *poly1* option. The AICc was calculated as in [33]. 

The pairings of FMS and S were defined by an arbitrary “proximity” relationship between the skills. The pairings were defined as a countermovement jump and high jump, standing long jump and long jump, and leap and hurdle transposition.

Provided that the performances’ relationships can be misleading (see [15]), we also considered the demonstrated movement pattern. The second step, then, considered whether participants with the best performances of the sport skills showed similar movement patterns at the fundamental skills. This would imply that those who reached higher levels of performance had to demonstrate something fundamental to reach these performances. For this, we performed, for each of the fundamental skills, the normalized dot product among participants (considering both the first and second principal components). The normalized dot product ranges from 0 to 1 and values above 0.9 are considered as high similarity (see [34]).

#### 2.4.5. Generalization and Adaptation

After the assessment of a potential proficiency barrier, we investigated whether we would find signs of generalization/adaptation between the performed movement patterns in fundamental and sport skills. For all cases, we ordered the participants in terms of the demonstrated performance of the sport skill.

First, we tested whether individuals’ “generalization” (maintenance of the fundamental movement pattern in the sport skill) was a function of the performance demonstrated in the sport skill. Generalization was measured using the normalized dot product between the same skill’s pairings used for testing the proficiency barrier. We compared the skills using the first and second principal components. Then, we calculated the Spearman’s *ρ* correlation to evaluate whether there was any relationship between the performance of the sport skill and the relationship among movement patterns.

Second, we aimed to compare whether generalization/adaptation of the movement pattern occurred, in general, for tasks with/without the presence of a proficiency barrier. We considered the first and second principal components of each task as a single vector and performed the normalized dot product between the fundamental and sport skills pairings. Then, we performed a Friedman’s ANOVA to establish whether tasks that showed a potential proficiency barrier were, indeed, the ones that required adaptation of the performed movement pattern.

Third, considering the same skill pairings, we tested whether the change in the number of components needed to perform the sport skills from the fundamental skill was a function of the skill reached for the sport skill. For this, we calculated the cumulative sum of variance accounted for each principal component and noted how many principal components were necessary to explain at least 90% of the data. Then, we subtracted the required components of the sport skills from the fundamental skills and correlated (using Spearman’s *ρ* correlation) this with the performance achieved for the sport skill.

Considering the current sample size, our analysis had a sensitivity for an effect size of 0.49 for the correlations (*Point Biserial Model*, power of 0.80, α of 0.05, and two tails).

## 3. Results

### 3.1. General Associations

The linear mixed effect models for the performance of each motor task showed that performance was always affected by age: countermovement jump (estimate: 0.05; *t* [260] = 12.99; *p* < 0.001); standing long jump (estimate: 5.82; *t* [248] = 3.64; *p* < 0.001); leap (estimate: 0.02; *t* [257] = 2.52; *p* = 0.012); high jump (estimate: 0.06; *t* [255] = 4.91; *p* < 0.001); long jump (estimate: 0.11; *t* [262] = 3.94; *p* < 0.001); and hurdle transposition (estimate: 0.03; *t* [260] = 4.00; *p* < 0.001). Thus, in all cases, the older the individual, the better the performance.

In a few tasks, trial also showed a significant effect: standing long jump (estimate: −3.73; *t* [248] = 2.11; *p* = 0.036) and hurdle transposition (estimate: −0.004; *t* [260] = 2.53; *p* = 0.012). Thus, for these two tasks, individuals showed a tendency toward a decrease in performance over the trials.

For the number of components required to account for 90% of the variance, we found a significant effect of task (*F* [5] = 88.58; *p* < 0.001). The pairwise comparisons (with Bonferroni’s correction) showed that the countermovement jump showed the smallest number of components (mean: 1.16; *p*-values < 0.050 against all other tasks), and that the high jump showed the largest number of components (mean: 3.22; *p*-values < 0.050 against all other tasks). All other tasks did not differ in between.

Table 2 shows the Spearman’s *ρ* correlations among the performances of all of the skills. From this, we can observe a high level of association among these skills.

Provided the largest effect of age on all tasks, we also calculated the Spearman’s *ρ* partial correlations among all tasks, controlling for the effect of age. Table 3 shows the Spearman’s *ρ* partial correlations among the performances of all of the skills. From this, we see that a large number of the associations observed before can be accounted for by age.

### 3.2. Proficiency Barrier Assessment

**Figure 2:**
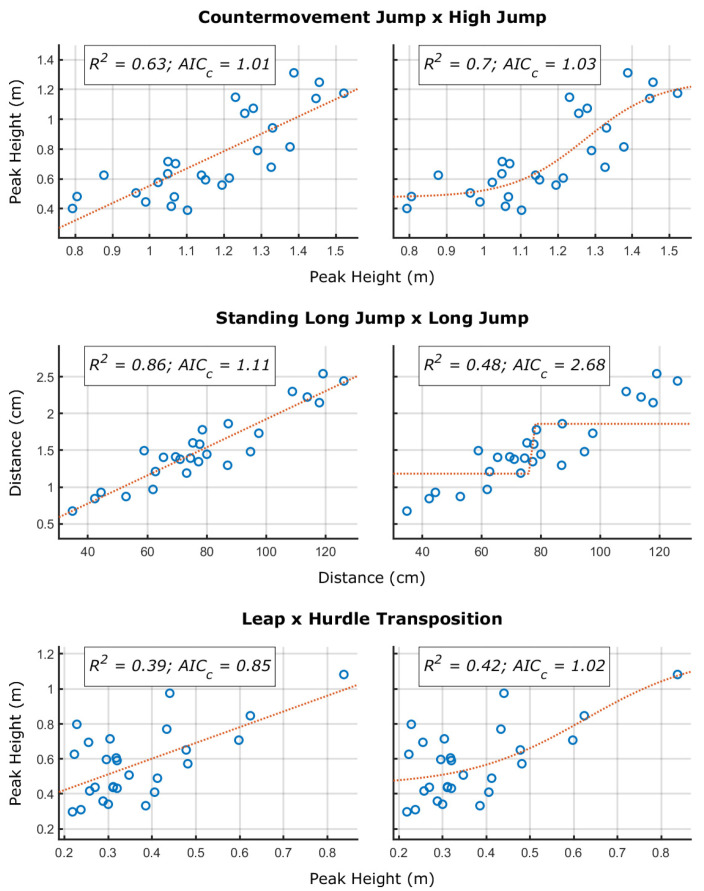
Sport skill performance as a function of the fundamental skill performance. Each circle represents an individual.

Figure 2 shows the sigmoidal and linear functions fitted to the relationship between countermovement jump and high jump, standing long jump and long jump, and leap and hurdle transposition. As shown in the figures, for all cases, the AICc was smaller for the linear function—despite a similar AICc between linear and sigmoidal functions for the countermovement jump and high jump pair.

**Figure 3:**
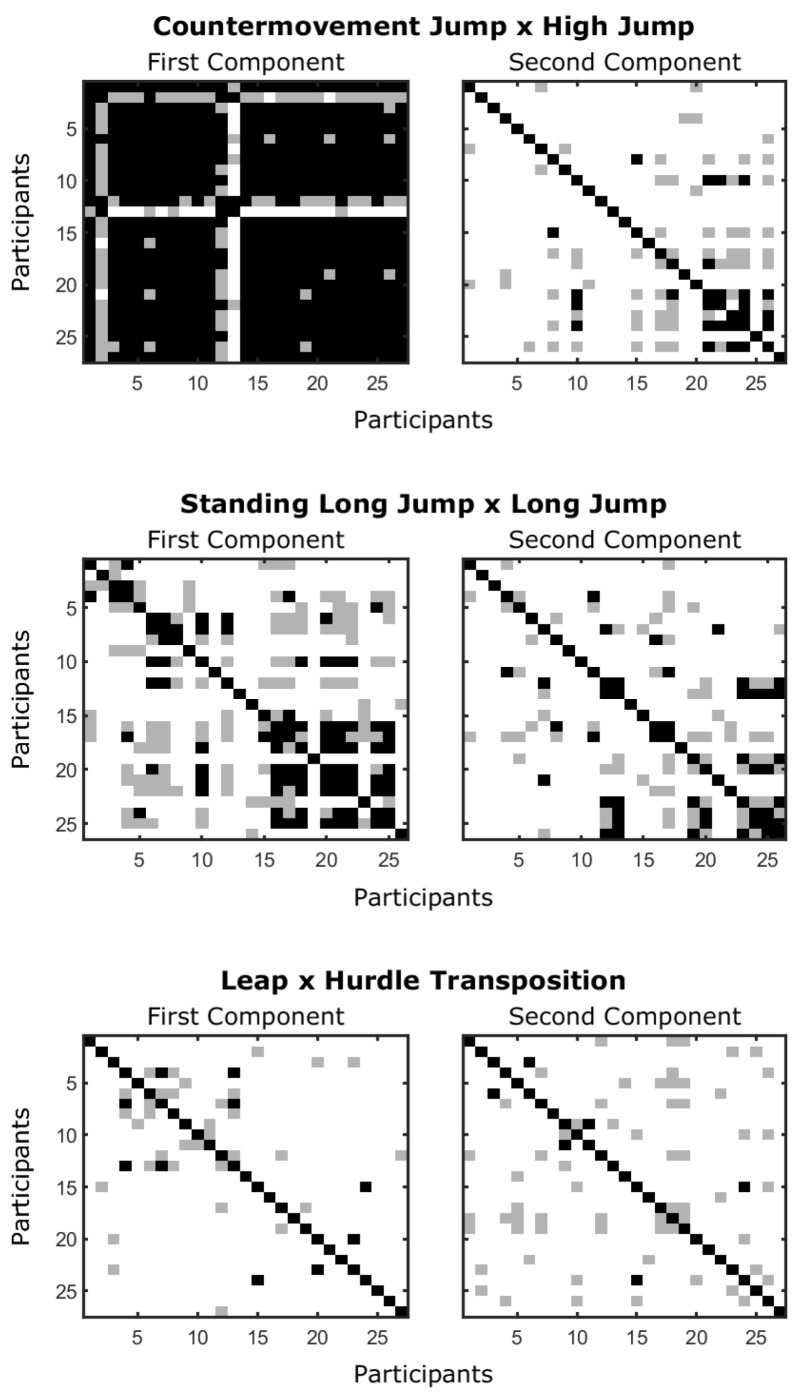
Similarity among individuals (normalized dot product) in their fundamental movement pattern (first and second principal components). Black squares mean normalized dot products higher than 0.9 and gray squares mean normalized dot products higher than 0.8. Individuals are sorted by their performance of the sport skill with higher values (lower right corner) meaning better performances of the sport skill.

From Figure 2, one would then suppose that there is no evidence of a proficiency barrier observing the pairings. Nonetheless, as stated, the issue must be considered also in terms of the movement pattern demonstrated. Figure 3 shows the between-individuals similarity (considering the normalized dot product) in the fundamental movement pattern with individuals sorted in terms of the performance demonstrated of the sport skill. 

As can be observed, for the countermovement jump, the first component is quite similar between almost all participants. Nonetheless, for the second component, only some individuals who showed better performances in the high jump showed large similarity between them. For the standing long jump, both the first and second components showed a cluster of similarity in the movement patterns for individuals with better performances in the long jump. On the contrary, for leap, there was no clear pattern of similarity that emerged.

From these results, we can infer a potential proficiency barrier in two fundamental x sport skills pairings: the countermovement jump and high jump, and the standing long jump and long jump. It is important to note that the performance relationship could demonstrate the sigmoidal relationship only for the countermovement jump and high jump pair and that similarities in the fundamental movement patterns were not necessary for high levels of performance (there are individuals with high performance and no similarity with other high performers). We discuss these issues in Section 4. 

### 3.3. Fundamental Movement Patterns and Performance of Sport Skills

**Figure 4:**
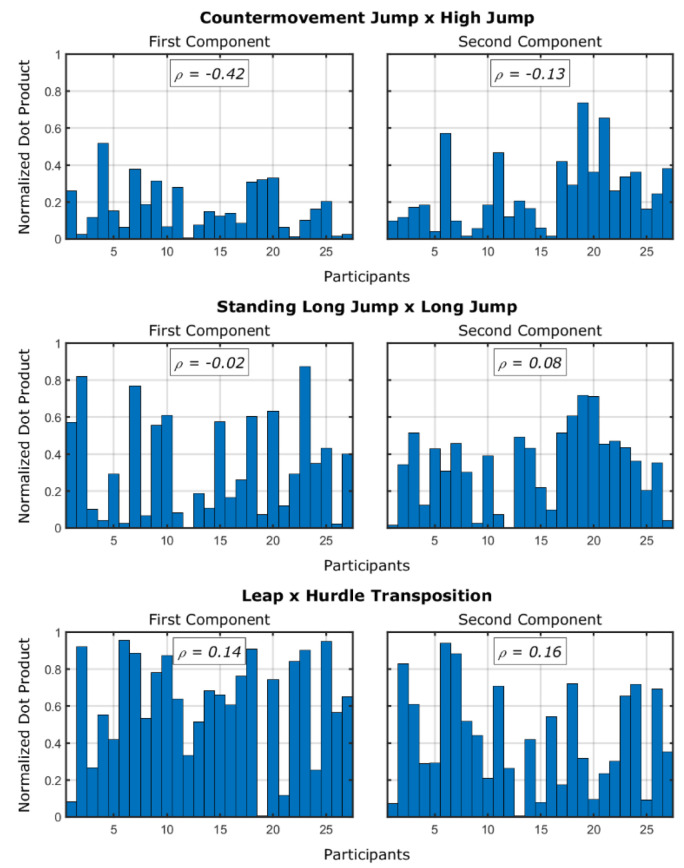
Similarities between the fundamental and sport skills by the same participant (normalized dot product) according to their movement patterns (first and second principal components). Individuals are sorted by their performance of the sport skill, with higher values meaning better performances of the sport skill. The *ρ*-values represent the Spearman’s *ρ* correlations between the normalized dot products and the performances of the sport skill.

Figure 4 shows the normalized dot product for the fundamental and sport skills pairings as a function of performance of the sport skills and the principal components considered. From the association between the normalized dot product and the performance of the sport skill, the only significant correlation is a weak association between the normalized dot product between countermovement jump and high jump (on the first component) with the performance in the high jump (*ρ* = −0.42; *p* = 0.029). This would imply that those who showed more generalization are the ones with worse results in the sport skill. Note, however, that the normalized dot product values were already low in this case.

It could be that the pairings showing the proficiency barrier were the ones that, in general, required adaptation. We found an effect of fundamental and sport skills pairing on the similarity of movement patterns (i.e., normalized dot product of the single vector encompassing both the first and second components between the fundamental and sport skills) (*F* [2] = 8.67; *p* = 0.013). The pairwise comparisons (with Bonferroni’s correction) showed that the countermovement jump and high jump pairing had lower normalized dot products compared to the leap and hurdle transposition pairing (*p* = 0.013). 

**Figure 5:**
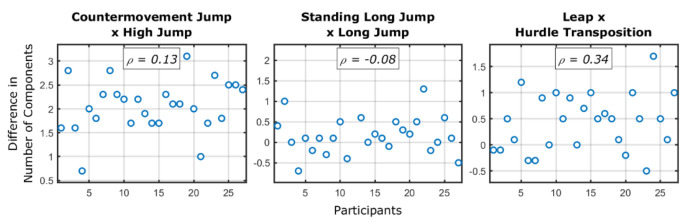
Difference between the fundamental and sport skills (per pairing) in the number of components required to explain at least 90% of the accounted for variance. Individuals were sorted by their performance of the sport skill, with higher values meaning better performances of the sport skill. The *ρ* values represent the Spearman’s *ρ* correlation between the difference in the number of components and the performance of the sport skill.

Figure 5 shows the difference between the number of components in the fundamental and sport skills pairings as a function of performance of the sport skills. As it is observed, there is no correlation between increase or decrease in the number of components between skills and the performance of the sport skills. As expected from the results in the general association section, the largest difference in number of components occurred for the countermovement jump and high jump pair.

## 4. Discussion

The increased capacity to act in new contexts given previous experiences is one of the cornerstones of human survival through life. Despite numerous claims about the specificity (e.g., [35]) and limited transfer (e.g., [36]) of motor skills, if learning was, indeed, limited to the condition being practiced, one would not have sufficient time to practice and learn all required skills and their variations to survive. Indeed, it is in the motor development and training literature that authors acknowledge the difficulty of reaching high levels of performance in a number of skills and, for this reason, place high importance on early experiences (see [2,4,6,37]). Nonetheless, empirical demonstrations of this importance are still lacking in a vast range of contexts. In fact, little is known about when and how a dependence on previous experiences would be observed. 

In the present paper, we investigated how fundamental skills relate to sport-specific skills in track and field. We based this on recent studies on the topic of the proficiency barrier ([9,14,15,16], see also [17]). These studies provide conflicting findings about the hypothesis that basic components of the so-called fundamental movement skills are necessary for learning more complex or specialized sport skills. Considering the potential different processes that might underlie the relationship between fundamental and sport skills (i.e., generalization and adaptation), our aim was to, first, investigate whether the proficiency barrier would be observed and, second, whether its occurrence was dependent on the required transfer process.

### 4.1. Proficiency Barrier

From our results, we inferred a proficiency barrier in the relationships between countermovement jump and high jump, and the standing long jump and long jump. In the former pair, both performance (despite slight support for a linear relationship) and movement pattern similarity (between-individuals) supported this inference. In the latter pair, only the movement pattern similarity supported the inference. This might have occurred provided the nonlinear (and redundant) relationship that movement patterns have with performance outcomes (as demonstrated in [15]). This reinforces that if there is a proficiency barrier between fundamental and specific skills, it is demonstrated in the movement kinematics rather than the outcome.

For the leap and hurdle transposition pair, we did not see any sign of a proficiency barrier. This might indicate that there is direct transfer from one to the other. This is a hard argument to hold as the correlation between the two (in terms of performance) was weak and nonsignificant when age was controlled for (however, see the limitations below). Additionally, one might expect that direct transfer may demonstrate a large degree of generalization. At least in terms of the aspects analyzed here (the coordination between joints), we did not find evidence for generalization. One could question whether the leap is fundamental for the hurdle transposition: although the leap requires increasing the step forward, the hurdle transposition requires an attempt to maintain the velocity forward while passing over the hurdle vertically. However, detailed descriptions of all pairs here could lead to similar arguments. As discussed in our limitations section, deciding what is fundamental to what is still an issue.

The two skill pairs supporting the proficiency barrier also showed distinct movement pattern similarity groupings (i.e., black squares in Figure 2). While only the second component showed the expected grouping for the first fundamental/sport skills pair, both the first and second components showed the expected grouping for the second pair (with groupings encompassing a different number of participants and performance levels). The fact that the first principal component of the countermovement jump did not differentiate the levels of performance in the high jump only shows that this movement component is either simple or necessary for the execution of the task. A closer look at the coefficients shows that, for the majority of participants, the first principal component captured the correlated flexion of ankle, knee, and hip—a coupling that they all showed to varying degrees. The second coefficient seems to be a compensatory movement of the knee and hip negatively related to the ankle—something that (1) is not necessary to perform the jumping and (2) seem to be a pattern that requires more practice.

The same logic can be applied to the relationship demonstrated in the first and second components between the standing long jump and long jump: the first component seems to describe an easier to acquire movement component while the second requires long-term practice to be demonstrated. The first component (of the similar group) represents a positive relationship between ankle, knee, hip, and thorax flexions while elbow and shoulder are extending (mostly present at the beginning of the movement). The second component (of the similar group) represents a positive relationship between knee, hip, and shoulder (present toward landing). Both seem to provide a basis for better outcomes in the long jump.

Another important outcome of the present analysis is that, despite the fact that we found common patterns in the fundamental skills that related to the performance of the sport skill for the two pairs, they were not *necessary* for better outcomes. That is, there were some individuals who did not show a similar pattern and had good performances of the sport skill. Thus, contrary to the *strict* proficiency barrier hypothesis [14], which postulates that only those who present proficiency in given components of the fundamental skill will be able to learn the more specific task, there seems to be other paths to learning. This possibility is what O’Keeffe and colleagues [17] showed when testing the transfer from an overarm throw to a dart throw and badminton overhead. They showed that the group who performed only the sport-specific skill (badminton overhead) did improve in the skill, despite having low proficiency in the overarm throw (a fundamental skill).

### 4.2. Generalization and Adaptation

This leads us to the second big topic of the current study: processes of transfer. From the dynamical systems approach to motor learning and development (see [21,38]), the initial condition of the system (learner) has a large influence on the process of change that the system will go through. From this point of view, learning will always demonstrate transfer effects given that the previous practice will always affect new learning events. The question is the degree of change that the new practice requires from what was previously learned (see [39]). If the new task’s demands are in line with the system capacities, there are greater chances of seeing generalization. If the new task’s demands require modifications, previous practice might offer a better starting point for exploration and change (which we would refer to as adaptation here). If previous practice is insufficient for dealing with the new task’s demands, one would observe a phenomenon similar to the proficiency barrier (see [23] for a similar line of thinking).

Our hypothesis was that generalization would be found when the proficiency barrier is absent. Our results did not fully support our hypothesis. For the countermovement jump and high jump, we have some support: (1) these two skills were the most different in terms of the required number of movement pattern components (indicating the need for adaptation); (2) all participants demonstrated lower similarity values when the fundamental and sport movement patterns were compared (indicating changes in their movement pattern); (3) those with better outcomes in the sport skill were the ones who were able to better change their first movement component; and (4) the clearest signs of a proficiency barrier were demonstrated in this pairing.

However, considering the standing long jump and long jump, individuals varied in their similarity—independent of the sport skill performance. Thus, it might be possible to show either generalization or adaptation and still succeed in the more specific skill. This is a clear example of the potential multiple paths of development. Additionally, the pattern of generalization/adaptation in the leap and hurdle transposition pairing was also quite variable—which reinforces the possibility of individuals succeeding through different paths.

We emphasize that transfer, despite long-term discussions on the issue (e.g., [40,41,42]), is still far from being understood. We envisage a range of processes (beyond generalization and adaptation) that must be encompassed under the term (e.g., “learning to learn” [43,44]) before definitive understanding of the potential hierarchies that exist in learning and development. In fact, for practitioners, transfer should be of primary concern as interventions (e.g., sports training, rehabilitation, and physical education) are usually a small set of activities planned to provide the largest impact on the maximum range of situations. Without any good ground on the principles of transfer, we see no pathway to design appropriate interventions.

### 4.3. Limitations

The first limitation of the present study is the arbitrary choice of fundamental and sport skills pairs. Indeed, one could claim that the standing long jump is fundamental for high jump as well (see Table 3 for a potential argument) or that other skills are fundamental rather than the ones chosen here. We have no firm theoretical argument against these possibilities rather than the potential kinematic similarity that these tasks share. In fact, to the best of our knowledge, we see no clear theoretical grounding to suppose that any task is more fundamental than any other in the literature. Even considering Newell’s [11] proposed fundamental skills (reaching, standing, and locomotor skills), the question is whether one needs to learn these skills first before attempting to learn skills that are “more complex”. Can one learn these skills *while* trying to learn more complex movement patterns? In the initial stages of development, the answer to these questions seems simpler. However, proposed sequences of motor skills to be acquired/practiced in late childhood, sports initiation, and even rehabilitation abound without proper principles to defend them.

The second limitation of the present study is the presence of age as a confounder. As age carries both growth and experiences effects (which can also interact), a superior performance could have occurred because one individual has more strength and, despite bad technique, showed better results than someone who demonstrates the inverse. This type of issue can only be accommodated with larger samples, more tests (to control for different physical abilities), or longitudinal studies. In the present study, as the main focus was on the relationship between movement patterns, we do not see this limitation as a major issue.

The third limitation of the present study is the superficial notion of “components” employed here. Previous studies on the topic utilized assessment based on movement criteria (e.g., [26]). These criteria are descriptions that might involve quantitative aspects (e.g., “the child approaches the ball in a straight path”, “the child bounces the ball three times without losing control”), relationships (e.g., “the left arm moves forward while the right arm moves backward”), and timing (e.g., “the lower and upper parts of the trunk face the target at the same time”). Nonetheless, the components extracted here refer only to correlated time-series—relationships—with no reference to quantity or timing. In other terms, our analyses—despite being more detailed and avoiding predefined movement pattern standards—might have been limited to a single dimension of the movement pattern. Thus, for the description of the components above, it was not that the rest of participants did not implement coordinated movements of the mentioned joints, it was just that they do it in ways that are not similar to others. Differences might have occurred in terms of the timing of the motion and the joints that actively participated in the task. Further developments are required to encompass these other dimensions in the type of investigation performed here.

## 5. Conclusions

As humans learn to perform new motor skills, their potential range of interactions with peers and activities in their contexts increase exponentially. All of this is a result of transfer processes in learning and development. The present study addressed whether the performances of the so-called fundamental skills demonstrated a “necessary” condition for good performances of the sport skills (i.e., the proficiency barrier)—specifically in track and field jump events—and whether such a relationship was dependent on the transfer process occurring. We found that despite the evidence favoring a proficiency barrier, the transfer processes are not related to it. This result seems to point to a multiplicity of paths in motor development.

## Figures and Tables

**Table 1:** Sample characteristics (age, height, weight, body fat percentage, and categories).

Age	Height (m)	Weight (kg)	Body Fat %
12.31 ± 3.19	1.51 ± 0.19	45.66 ± 15.60	19.40 ± 6.52
Age Categories (*n*)			
Under 10		5	
Under 12		5	
Under 14		5	
Under 16		7	
Under 18		3	
Under 20		2	

**Table 2:** Spearman’s *ρ* correlations among all skills.

	CMJ	SLJ	L	HJ	LJ
SLJ	0.91				
L	0.36 ^ns^	0.41			
HJ	0.78	0.79	0.45		
LJ	0.83	0.86	0.52	0.77	
HT	0.71	0.83	0.38 ^ns^	0.73	0.78

CMJ: countermovement jump; SLJ: standing long jump; L: leap; HJ: high jump; LJ: long jump; HT: hurdle transposition. ^ns^ Nonsignificant correlation.

**Table 3:** Spearman’s *ρ* partial correlations among all skills, controlling for age.

	CMJ	SLJ	L	HJ	LJ
SLJ	0.55				
L	0.17 ^ns^	0.28 ^ns^			
HJ	0.34 ^ns^	0.42	0.34 ^ns^		
LJ	0.41	0.58	0.46	0.43	
HT	0.25 ^ns^	0.63	0.24 ^ns^	0.44	0.52

CMJ: countermovement jump; SLJ: standing long jump; L: leap; HJ: high jump; LJ: long jump; HT: hurdle transposition. ^ns^ Nonsignificant correlation.

## Data Availability

The data and scripts necessary to generate the results of the present study are all available at https://osf.io/bgxa8 (access date: 3 February 2024).
